# Effect of Resistance Training on Body Composition, Hemodynamic Parameters and Exercise Tolerance among Patients with Coronary Artery Disease: A Systematic Review

**DOI:** 10.3390/healthcare11010131

**Published:** 2022-12-31

**Authors:** Abhishek Sharma, Nidhi Sharma, Sakshi Vats, Mansi Jain, Aksh Chahal, Faizan Z. Kashoo, Ali Hakamy, Ramzi Abdu Alajam, Mohammed M. Alshehri, Mallela Bharath Kumar, Ramya Ramasamy Sanjeevi, Fawwaz Alwadaani, Mohammad Abu Shaphe

**Affiliations:** 1 Department of Paediatric and Neonatal Physiotherapy, Maharishi Markandeshwar Institute of Physiotherapy and Rehabilitation, Maharishi Markandeshwar (Deemed to be University), Mullana 133207, India; 2 Department of Neurological Physiotherapy, Maharishi Markandeshwar Institute of Physiotherapy and Rehabilitation, Maharishi Markandeshwar (Deemed to be University), Mullana 133207, India; 3 Department of Musculoskeletal Physiotherapy, Maharishi Markandeshwar Institute of Physiotherapy and Rehabilitation, Maharishi Markandeshwar (Deemed to be University), Mullana 133207, India; 4 Department of Physical Therapy and Health Rehabilitation, College of Applied Medical Sciences, Majmaah University, Al Majmaah 11952, Saudi Arabia; 5 Respiratory Therapy Department, Faculty of Applied Medical Sciences, Jazan University, P.O. Box 114, Jazan 45142, Saudi Arabia; 6 Physical Therapy Department, College of Applied Medical Sciences, Jazan University, P.O. Box 114, Jazan 45142, Saudi Arabia

**Keywords:** body composition, coronary artery disease, exercise tolerance, hemodynamics, resistance training

## Abstract

Background: Effectiveness and safety of Resistance Training in treating various Cerebrovascular Disease diagnoses have drawn attention in recent years. Patients suffering with coronary artery disease should be offered individually tailored Resistance Training in their exercise regimen. Resistance Training was developed to help individuals with their functional status, mobility, physical performance, and muscle strength. Objective: The objective of this review was to collect, summarize and present information on the state of science focusing on usefulness, viability, safety and efficacy of Resistance Training in treating coronary artery disease and enhancing the aerobic capacity and improving overall health-related quality of life. Methods: The review is prepared in accordance with Preferred Reporting Items for Systematic Review and Meta-Analyses guidelines. Searches were conducted in Cochrane Library, PubMed/MEDLINE, PEDro and Scopus database. PEDro scale was used for methodological quality assessment of included studies. Two independent reviewers determined the inclusion criteria of studies by classifying interventions based on core components, outcome measures, diagnostic population and rated the quality of evidence and strength of recommendations using GRADE criteria. Results: Total 13 studies with 1025 patients were included for the detailed analysis. Findings emphasize the importance of assessing effectiveness and safety of Resistance Training in individuals with coronary artery disease. Patient specific designed exercise programs as Resistance Training targets at enhancing patients’ exercise tolerance, improves hemodynamic response and muscular strength with reduction in body fat composition. Conclusion: Resistance Training is an effective exercise that should be incorporated to counteract the loss of muscle strength, muscle mass, and physiological vulnerability, as well as to combat the associated debilitating effects on physical functioning, mobility and overall independence and Quality of Life during rehabilitation of patients with coronary artery disease.

## 1. Introduction

One of the most common cardiovascular disorders impacting people worldwide is Coronary Artery Disease (CAD). It has been established that this illness is the main cause of death in both developed and developing nations. Development of cardiovascular disease (CVD) is high following to lifestyle, environmental, and hereditary factors [1]. More people die from cardiovascular illness than from any other cause worldwide, where CAD accounts for over 40% of deaths [2]. Atherosclerosis, a lifetime process which can commence in childhood as fatty lesions and develop to flow-limiting stenosis of major epicardial coronary arteries, eventually manifests as angina and/or myocardial infarction, is one of the key pathological processes contributing to CAD-related morbidity [3].

Risk factors for CAD associated with atherosclerosis in the adults account for patients aged over 35 years, but it is still unclear whether the presence of an atherosclerosis lesion in a young patient can also be a risk factor for the same [4]. Modifiable risk factors linked to CAD are smoking, diabetes mellitus, high blood pressure that can be altered by modifying lifestyle, maintaining normal body weight and consuming a healthy diet and cessation of alcohol and its associates [5].

CAD can be managed by medical and surgical treatment methods. Medical treatment is comprised of ranolazine, nitrates, β-blockers, calcium antagonists, and antiplatelet few therapeutic medications to treat symptomatic angina related to CAD [6]. Invasive or surgical approaches to treat CAD are Coronary Artery Bypass Grafting (CABG), Angioplasty and Stent placement [7]. Till date continuous aerobic exercise at moderate intensity has been the mainstay for cardiac rehabilitation, with guidelines for its usage whenever applicable [8]. New evidence emphasized safety and efficacy of High-Intensity Interval Training (HIIT) as it positively impacts parameters such as cardiovascular capacity and endothelial function, left ventricular functioning and health related quality of life (QoL). A Rating of Perceived Exertion (RPE) between 15 and 18 with an intensity peak of >85% VO_2_ characterize this type of exercise. Alternating brief, lower-intensity workouts with rest periods may be advantageous for older adults [9].

Beside the above stated treatment, physiotherapy plays a vital role in decreasing risk of CAD and its associated factors. It comprises of Aerobic Exercises (AE), Endurance Training (ET) [10], Resistance Training (RT) [11], water-based exercises (WBE) [12], HIIT [13]. Thus, the purpose of this review is to summarize the effect of RT session on functional capacity, physiological parameters (blood pressure, heart rate), and health related QoL in patients with CAD.

## 2. Materials and Methods

### 2.1. Protocols and Registration

This review was conducted in accordance with the Preferred Reporting Items for Systematic Reviews and Meta-Analyses (PRISMA) standards. The study was registered with PROSPERO, an international prospective registry of systematic reviews with ID CRD42022375751.

### 2.2. Criteria for Considering Studies 

The following standards were to be met by published studies to be included in present review: Randomized Clinical and randomized controlled trials, Interventional approaches: Studies in which patients with CAD received RT as an intervention; Study participants; Patients with verified diagnosis of CAD were only included. The included studies looked at how RT has been used to influence hemodynamic parameters, body composition and exercise tolerance. Excluded from the review were non-human trials, research involving patients with neurological conditions such as stroke, etc., and interventions that predominantly fall under the purview of another profession, such as pharmacotherapies, psychotherapy, speech therapies, etc.

### 2.3. Search Strategies and Data Resources

Authors searched databases like Cochrane Library, PubMed/MEDLINE, PEDro and Scopus. We carried manual searches using the following conditional keywords: Resistance exercises AND coronary artery disease, Resistance exercises AND hemodynamic parameters, Resistance exercise AND exercise tolerance, Resistance exercises AND exercise tolerance AND coronary artery disease. Boolean logical operators were employed to combine the specified phrases (OR, AND, NOT). In addition, we manually searched most of the reference found in the chosen publications. Rayyan web software was used to examine each reference [14].

### 2.4. Data Extraction

Data extraction was done using a method akin to screening. An Excel pilot sheet with three primary tabs—baseline characteristics tab, tab for expected results, and third tab for quality assessment—was created by a senior author (Table 1 and Table 2). Researchers with competence in training of literature reviews independently reviewed the chosen articles. Two other researchers reviewed the studies abstract and title of the studies. A third reviewer resolved any differences. Full-text versions of the articles chosen in the previous stage were read and compared to the eligibility requirements. A reviewer resolved any differences. When possible, we got more unpublished data from the study’s authors.

### 2.5. Methodological Quality Assessment

The included research articles were qualitatively assessed using the PEDro scale. The internal validity of the selected articles was assessed using PEDro scale, which can be obtained at https://pedro.org.au/scale_item.html#scale_1. (Last accessed on 10 November 2022). Studies examining the efficacy of physical therapy treatments can be found in a database called PEDro. To assess the reliability and superiority of the methodology used in randomized clinical trials, an 11-item scale was developed. An item should receive a score of “1” for a favorable response if it has been considered, and a score of “0” if it has not. The total of all yes responses is used to calculate the result (Table 3).

### 2.6. Collecting, Summarizing and Reporting Results

The data has been compiled, synthesized and conducted to both quantitative and qualitative analysis. In the quantitative section, we provide a numerical description of the scope, nature and distribution of the research that this study examined. According to Khan KS steps for systematic review, we provided a narrative review of the available data for the qualitative analysis that addressed the study questions and placed an emphasis on the implications in a wider context. We finished up by addressing potential future research areas [25].

## 3. Results

### 3.1. Selection of Studies

**Figure 1:**
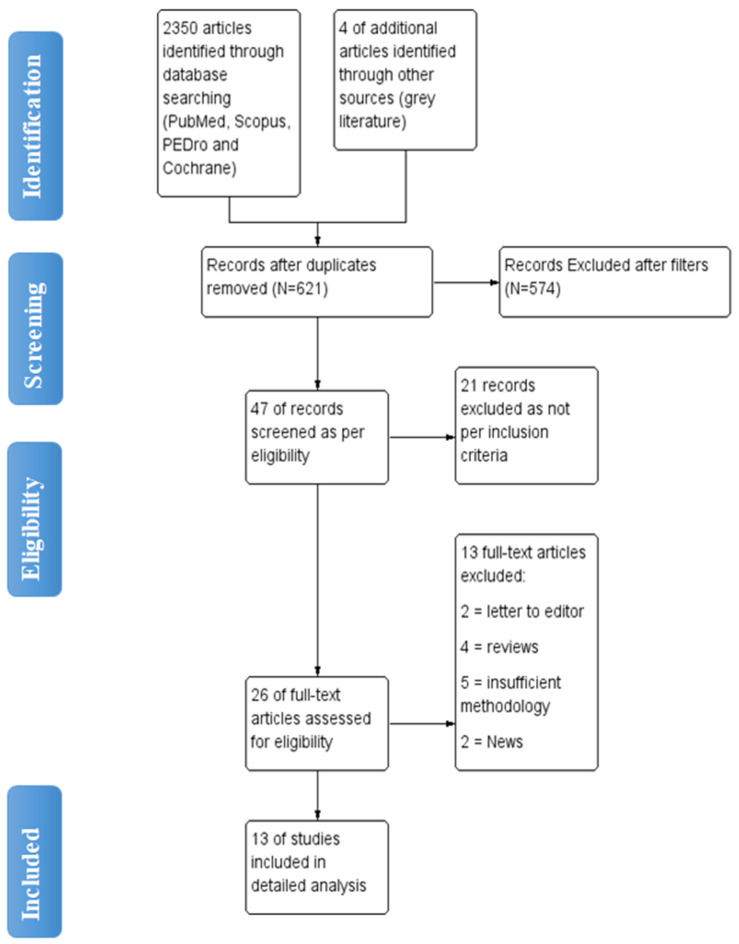
Flow diagram of article selection for review.

The initial search yielded 2350 articles from various electronic database search, with 4 articles were found through grey literature search. Duplicates were found and eliminated using EndNote Software https://endnote.com/ Philadelphia, USA leaving with total 621 articles. (Last accessed on 10 November 2022). 26 articles were found to be appropriate for full-text screening after the titles and abstracts of articles were reviewed. Total 13 studies with 1025 patients were included for detailed analysis (Figure 1).

### 3.2. Risk of Bias Assessment

**Figure 2:**
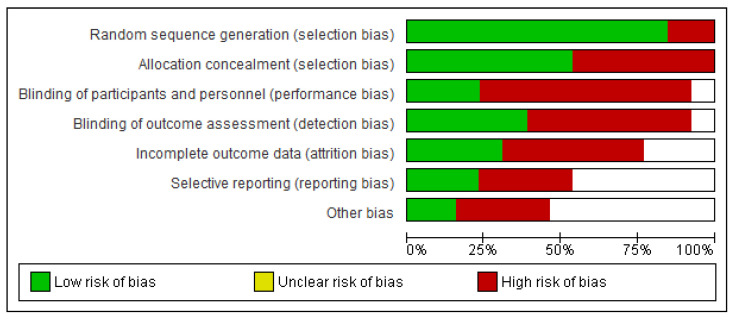
Risk of bias graph.

**Figure 3:**
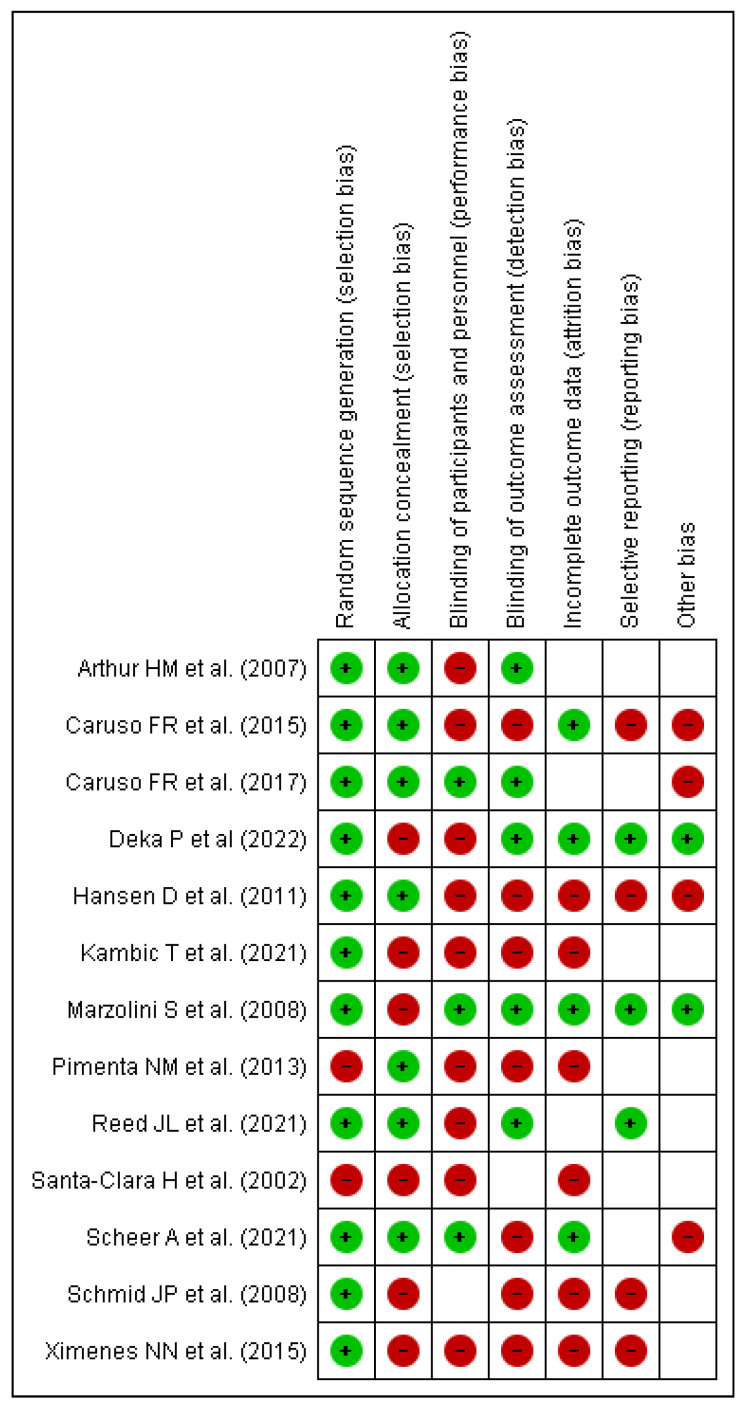
Risk of bias summary of included articles for review [10,11,12,13,16,17,18,19,21,26,27,28,29].

In nearly all studies, concealment of allocation produced a low risk of bias in the creation of the random item sequence, but blinding of result evaluators posed a high risk of bias This is expected to occur because the authors did not disclose or carry out these steps in the trials. For the blinding items of participants and experts, there was a high or unknown chance of incomplete results and various types of bias. Only the item presenting a selected outcome had the minimum risk of bias, as shown in Figure 2 and Figure 3. This is because the study methodologies and major outcomes were fully disclosed.

### 3.3. Main Findings

Inclusion of RT bearedes early Aerobic Endurance Training (AET) intervention in patients with CAD is medically safe and has beneficial impact on wide range of vital health parameters. In addition, combined AET and RT is well tolerated in patients with CAD and brings in additional pronounced physiological adaptations than AET alone.

### 3.4. Impact on Hemodynamic and Metabolic Parameters

In a study combining low-intensity RT along with AET, post-intervention showed significant increase in plasma HDL-c content (with 4.5 mg/dL + 6.4) vs AET (with 0.6 mg/dL + 4.3) (*p* < 0.05). Change in plasma HDL-c content positively correlated with change in VO_2_ peak (r = 0.32, *p* < 0.05) and plasma CRP level (r = −0.46, *p* < 0.05) [15]. RT, when given along with aerobic exercises resulted in rise of maximal and submaximal load tolerance (*p* < 0.01) with reduced hemodynamic response (*p* < 0.01) and decrease in blood lactate [16]. As Ventricular Threshold (VT) is a significant exercise parameter in CAD patients, the combined exercise group (AET with RT) increased VT by 32.8% compared to 22.6% in the AET and a 14.1% decrease in the control group. Improvement was greater in the combined exercise group compared to other groups [17].

### 3.5. Impact on Exercise Tolerance and Peak Oxygen Consumption

Peak oxygen consumption is one of the vital predictive indicators when dealing patients with CAD. Improvement of 1 mL/kg/min is associated to a 9–10% reduction in cardiovascular mortality. An interventional training program combining AET with RT, led to remarkable increase of exercise tolerance (1.8 ± 0.3 vs. 2.0 ± 0.4 W/kg; *p* < 0.001) and peak oxygen consumption of (22.8 ± 4.5 vs. 25.9 ± 5.5 mL/kg/min, *p* < 0.001) [18].

Effects of RT when applied early following coronary artery bypass grafting revealed absence of exertion to be induced by RT on pulmonary function. The intervention maintained functional capacity till the discharge from hospital which is measured by percentage of predict distance in 6 Minute Walk Test (54.1 ± 22.7% vs. 52.5 ± 15.5%, *p* = 0.42), while control group presented with a significant decrease (59.2 ± 11.1% vs. 50.6 ± 9.9%, *p* < 0.016) [10].

### 3.6. Impact on Body Composition

The total body lean tissue mass tended to increase with greater magnitude with RT (with 0.8 kg + 1.1) when compared to AET (with 0.0 kg + 1.4) (*p* = 0.07) [10]. There was pronounced reduction in total percent body fat in the group receiving RT along with AET. No changes were seen in the group receiving AET alone. Regional analysis reveals a significant decrease in percentage of arm, trunk and leg body fat in the former group (*p* < 0.05) [24]. Of the total mean fat mass loss in the groups receiving RT along with AET, 73% was from the trunk region in both groups [11]. Combined training brings forth a greater reduction in body fat percentage and produces a regional fat loss with respect to risk for both, metabolic and cardiovascular disease [11].

Body Fat (BF) content and its distribution in CAD patients following a 1-year combined AET and RT program revealed significant reductions in all analyzed BF depots, including total BF (21.60 + 6.00 vs. 20.32 + 5.89 kg, *p* < 0.01), percentage of total BF (27.8 + 5.5 vs. 26.4 + 5.4%, *p* < 0.05) and abdominal fat (2.95 + 1.06 vs. 2.75 + 1.10 kg, *p* < 0.05). In contrast, control group showed significant increase in appendicular fat (7.63 + 1.92 vs. 8.10 + 2.12 kg, *p* < 0.05) [19].

### 3.7. Impact on Muscle Strength and Endurance

Upper and lower body muscular endurance increased significantly in the combined training group (*p* < 0.05), but no impact through AET. Multiple set RT performed in combination with AET was more beneficial in yielding improvement in total body, arm and leg lean mass accumulation than single-set RT/AET, or AET alone [11]. On comparing the high load (HL) and low load (LL) resistance exercises (RE), both HL- and LL-RE were found to be safe and well-tolerated in patients with CAD. Compared with baseline, all the hemodynamic parameters increased during the exercise and returned to baseline levels after both HL-RE and LL-RE with exertion being higher only after the first set of HL-RE when compared with LL-RE [12].

### 3.8. Impact on Sympathetic System Activity and Left Ventricular Remodeling

An 8-week program designed LL-RT demonstrated significant decrease in mean heart rate in training group thereby improving heart rate variability. Reduction in Heart Rate Index in the training group indicates a potential decrease in the sympathetic activity and increased parasympathetic nervous activity indicating an important clinical benefit in CAD patients [24]. According to the findings of another study, early combined AET/RT program after a myocardial infarction, does not lead to negative remodeling of the Left Ventricle, compared with AET alone. Using MRI as the imaging technique, researchers observed RT to be safe, and no arrhythmias or cardiac symptoms occurred [17].

### 3.9. Psychological Benefits and Quality of Life

Cardiovascular rehabilitation poses positive psychological effect on patients. It restores their lost confidence, alleviates fear of a physical load, allows them to return into active life and eliminates a serious risk factor—physical inactivity [18]. A 6-month study with aim to determine impact of supervised combined strength and AT for women post CAD showed statistically significant improvements in physical quality of life (*p* = 0.0002), self-efficacy for stair climbing (*p* = 0.0024), lifting (*p* < 0.0001) and walking (*p* = 0.0012) [16].

## 4. Discussion

Although articles included in the present systematic review include only resistance exercises, continuous or interval methods used to perform exercise varied, which may have contributed to the heterogeneity in the results. However, there were not enough studies to conduct a meta-analysis using various exercise methods. Early AET with RT has a positive impact on verified crucial health indicators in CAD patients. This was the conclusion: In contrast to AET, combined AET and RT significantly increased plasma HDL-c levels. 

Despite a propensity to bigger gain in lean tissue mass, adding RT to an AET intervention did not help to boost the clinical effects [10]. There are reasons contributing to lack of significant clinical effect including, presence of numerous clinical benefits from AET, brief duration of the intervention program, lack of a sufficient variety of resistance muscle exercises, and/or low exercise intensity during resistance muscle training. Clinical advantages of AET with diverse variation were attained such as, increase in muscle strength, submaximal and maximal exercise performance and a reduction in the bulk of adipose tissue [30].

In contrast to AET alone (5 sessions per week) sessions of RT exercise resulted in equivalent or greater changes in cardiovascular fitness (VO_2_ peak), as well as notable improvements in muscular strength, local muscle endurance, lean mass accretion and body fat percentage in patients with CAD patients. Despite performing about 30% few aerobic exercise sessions, combined AET/RT training (single or multiple sets) produced similar gains in VO_2_ peak to AET alone. Given the connection between VO_2_ peak and survival in CAD patients [31] as well as the relatively low compliance rates, it is preferable to maximize increases in VO_2_ peak during cardiac rehabilitation [15].

Reductions in body weight, total body fat, and visceral adipose tissue are among the advantages of doing resistance training. Even though the difference in weight and fat loss is only a few kilograms, decrease in visceral fat is expected to improve patients’ cardiometabolic health. Importantly, even individuals who lost little to no weight showed reduction in visceral fat [11]. Further increase in muscle mass was observed by gradually increasing the dose of RT. During dynamic RT involving large muscle groups the ventrolateral medullar regions are responsible for feedback to the sympathetic nervous system, which causes information on an increase in sympathetic discharge to the cardiovascular system. The sympathetic nervous system mediates vasoconstriction in inactive muscles and visceral areas, facilitating the mechanical and metabolic changes stimulating type III and IV muscle fibers that are responsible for this feedback [32].

Findings also projects, following completing RT, cardiorespiratory indicators and indicators of exercise tolerance improve significantly, which is an important indicator regarding risk factor for CAD. Consistent, long-term aerobic exercise reduces blood pressure, blood sugar, cholesterol and abdominal obesity [33]. Regular exercise increases venous tone, lowers blood pressure, decreases resting and stress heart rates, all of which have good impact on an individual’s ability to function as normal. Additionally, it results in an increase in myocardial contractility [34]. After completing the training program, a trend toward lower resting heart rate and systolic as well as diastolic blood pressure was seen in terms of hemodynamics [35].

Physiological parameters at submaximal intensities reveal a substantial decline particularly in blood lactate concentration. These results point to an improvement in muscular endurance. This may have an advantageous effect on activities of routine daily life. Nevertheless, the method through which these parameters improve is not fully understood; such vasculature changes could potentially explain, as a result, an increase in muscle blood flow, improvements in lactate clearance, skeletal muscle changes, and oxidative capability following exercise [36]. 

An acute cardiovascular imbalance may be the cause of the drop in plasma volume that is observed during the recovery phase of RT, which would provide a physiological explanation for this phenomenon [37]. The blood entering the interstitial cellular space, which alters the sensitivity of the arterial baroreflex to maintain the variations in blood pressure brought on by a reduction in stroke volume, is cause of this imbalance (a consequence of an increase in heart rate after resistance exercise) [38]. As a result, greater metaboreceptors and mechanoreceptors are activated, resulting in sufficient blood flow to support the working muscles’ metabolic needs [39]. There may also be an increase in peripheral vascular resistance in arteries supplying visceral organs when reallocated blood travels to the active muscles during the recovery period in patients [26].

Greater cardiac sympathetic modulation activation and cardiac parasympathetic modulation withdrawal result from higher training volume. To put it another way, larger resistance training volume causes a greater magnitude of cardiac sympathetic modulation activation and cardiac parasympathetic modulation withdrawal than lower resistance training volume [40]. On the other hand, prior research has shown that, in contrast to a high volume of moderate- or low-intensity resistance training, a low volume of high-intensity resistance training significantly enhances strength, muscle size, force output, and pace of force development [41].

RT markedly raise Heart Rate Volume (HRV) indices. Rise in the vagal modulation indices (RMSSD and SD1) suggest RT program to have a significantly altered parasympathetic cardiac regulation. The possible impact of parasympathetic indices can be explained by a training-induced increase in baroreceptor activity [42]. Varied level load (Low Load vs. High Load) induces physiological reactions in HR and BP that are similar. With either type of RT, patients had no significant issues, and the second and third sets of RT produced equivalent RPE [20]. Authors recommend future research to plan a comparison commonly employed oscillometric techniques with continuous hemodynamic response monitoring during RT in CAD patients as well as in patients with other cardiovascular conditions, such as heart failure, who potentially benefit from RT as part of their Cardiac Rehabilitation [13].

The main recommendations across the globe support RT as an important non-pharmacological therapy for individuals suffering from chronic conditions [43]. In three Norwegian cardiac rehabilitation clinics, 4846 patients with coronary artery disease participated in structured high-intensity interval training and moderate-intensity training and the risk of cardiovascular events were found to be low [27]. High-intensity interval training is a simple and safe technique that is advantageous for people with CAD even though the overall level of evidence is moderate [28] In patients with heart failure, no fatalities were linked to any type of exercise therapy [29].

## 5. Clinical Application

Application of low-intensity resistance muscle training contributed to a considerable improvement in blood HDL-c content and a tendency to a greater increase in lean tissue mass. A desirable regional fat loss was measured in the trunk region (73%), thereby reducing risk for both cardiovascular and metabolic diseases. Furthermore, a similar increase in VO_2_ peak and muscle strength were observed, thereby potentially improving the muscles’ capacity for oxygen flow. Incorporating low intensity-high repetition resistance training presented with beneficial adaptations, indicating lower cardiovascular stress at a submaximal exercise intensity. 

RT led to a significant decrease in the blood lactate concentration leading to an increase in the muscle endurance, consequently, causing a positive impact on activities of daily living among patients with CAD. RT exercises have shown a significant improvement in physiological parameters in CAD patients, showing evident gains in muscle strength, local muscle endurance and reduction in body fat. High and low intensity resistance training demonstrated improvement in cardio-respiratory function by increasing type IIa muscle activity, improving GLUT4 translocation in skeletal muscles, thereby restoring metabolic flexibility. In addition, an increase in the energy expenditure and excess post-exercise oxygen consumption were also seen. Thus, the addition of RT yields more benefit to cardiovascular parameters of exercise performance, skeletal muscle function, and body composition compared to AET alone.

## 6. Limitations

The literature on the effect of RT on body composition, hemodynamic parameters, and exercise tolerance in patients with CAD is synthesized in the present review. There are a few limitations to address. First, we did not evaluate the impact of exercise based on the duration of the resistance training program. Even though program’s duration varied greatly in the included studies. Second, we did not evaluate the impact of exercise volume over the course of the week. This question was not answered by any of the included studies in the current systematic review. Small sample sizes in some of the included studies may have had an impact on our findings.

## 7. Conclusions

In CAD patients, RT is better tolerated and produces more profound physiological changes than AET. Even though prescribing more than one set of RT may result in decreased adherence to the recommended number of sets, it may\increase factors that influence VO_2_ peak, VT, reduced body endurance and muscle mass in a heart population. Despite a 28% reduction in the actual AET training stimulus, the combination of RT and AET results in higher gains from the cardiovascular endpoints of physical performance, skeletal muscle function and body composition than AET alone. Findings from this systematic review vehemently support use of multiple-set RT for patients requesting a higher RT stimulus as well as a combined training intervention in CAD patients.

## Figures and Tables

**Table 1:** Demographic characteristics of articles addressing the use of resistance exercises in coronary artery disease patients.

Author	Country	Research Outline	Total Participants	Mean Age (Years)	Time of Assessment
Hansen, D. et al. [10] 2011	Belgium	Prospective randomized controlled trial.	60 Patients	AET = 58.9 ± 0.2 RMT = 60.4 ± 8.9	Pre and post treatment
Caruso, F.R. et al. [11] 2015	Brazil	Randomized controlled clinical trial	28 Patients	Training group = 61.3 ± 5.2 Control group = 61 ± 4.4	Pre and post treatment
Scheer, A. et al. [12] 2021	Australia	Parallel group, randomized controlled trial	52 Patients	WEX = 66 ± 8 GEX = 67 ± 8 Control = 71 ± 5	Pre and post treatment
Reed, J.L. et al. [13] 2021	Canada	Randomized Controlled Trial	135 Patients	MICT = 60 ± 7 NW = 61 ± 8 HIIT = 61 ± 7	Pre and post treatment
Marzolini, S. et al. [15] 2008	Canada	Randomized controlled trial	53 Patients	AT = 57.9 ± 2.6 AT/RT1 = 60.9 ± 2.3 AT/RT3 = 62.7 ± 2.7	Pre and post treatment
Santa-Clara, H. et al. [16] 2002	Portugal	Randomized controlled trial	40 Patients	Combined training group = 55 ± 10 Aerobic training group = 57 ± 7 Control group = 57 ± 11	Pre and post treatment
Kambic, T. et al. [17] 2021	Slovenia	Randomized, crossover clinical trial	43 Patients	61 ± 10	Baseline, in between and post treatment
Caruso, F.R. et al. [18] 2017	Brazil	Randomized controlled trial	20 Patients	61.1 ± 4.7	Pre and post treatment
Ximenes, N.N. et al. [19] 2015	Brazil	Randomized controlled trial	34 Patients	Intervention group = 59.9 ± 7 Control group = 61.8 ± 6.7	Pre and post treatment
Deka, P. et al. [20] 2022	Spain	Randomized controlled single-blinded trial	90 Patients	HIIT + R = 69.2 ± 4.18 UC = 69.27 ± 5.68	Pre and post treatment
Arthur, H.M. et al. [21] 2007	Canada	Prospective two group, randomized controlled trial	282 Patients	Not specified	Pre, in between and post treatment
Schmid, J.P. et al. [22] 2008	Switzerland	Prospective randomized, controlled study	38 Patients	ET/RT = 54.7 ± 9.4 ET = 57 ± 9.6	Baseline, at 3rd month and 12th month
Pimenta, N.M. et al. [23] 2013	Lisbon	Randomized controlled trial	44 Patients	CET = 57 ± 12 Control group = 58 ± 11	Pre and post treatment

Abbreviations: AT = Aerobic Training, AET = Aerobic Endurance Training, RMT = Resistance Muscle Training, RT = Resistance Training, ET = Endurance Training, CET = Combined Aerobic and Resistance Exercise Training, GEX = Gym-based Exercise, WEX = Water-based Exercise, HIIT + R = High Intensity Interval Training and Resistance Exercise, UC = Usual Care group, NW = Nordic Walking, MICT = Moderate to vigorous intensity continuous training.

**Table 2:** Presentation of articles in line with objectives and main findings regarding exercise therapy in coronary heart disease patients.

Author/Year	Objective of the Study	Intervention	Outcome Measures	Results	Conclusion
Hansen, D. et al. [10] 2011	Evaluate the efficacy of aerobic training in combination of lower limb low intensity resistance training in patients with coronary artery disease.	Aerobic training group = Aerobic training for 3 times in a week for 8 weeks. Combined group = Limbs along with aerobic exercises 3 times a week for 8 weeks.	Peak oxygen intake Lean tissue mass Muscle strength	In comparison to AET, RT tends to increase the total body lean tissue mass with a higher magnitude (*p* = 0.07), and blood high-density lipoprotein cholesterol content with a considerably greater magnitude (*p* < 0.05).	Low-intensity RT during early aerobic endurance training in individuals with coronary artery disease accounts for a substantial increase in blood high-density lipid cholesterol content, along with tends to alter lean tissue mass.
Caruso, F.R. et al. [11] 2015	The effect of resistance training on improving heart variability in patients with coronary heart disease.	Resistance training group = Session of resistance training along with aerobic training for a total duration of 1 h, 2 times a week for 8 weeks. Usual care group = In this group patients were provided with a session composed of warm-up, aerobic exercises, stretching and cool-down of 1 h, 2 times a week for 8 weeks.	Heart rate Muscle strength Endurance	Significantly increase in RMSSD and SD1 indices in the resistance training group after 8 weeks of training session.	Significant improvement in heart rate volume, muscle strength and endurance in coronary artery disease patients.
Scheer, A. et al. [12] 2021	Effect of water-based circuit training on body fat, fitness and leg strength in patients with stable coronary artery disease.	Water based exercise group = Circuit of light aerobic and stretching exercises, with an alternative session of aerobic exercises with resistance exercises 60 min, 3 times a week 12 weeks. Gym based exercise group = Aerobic exercises and resistance exercises were given to the patients 60 min, 3 times a week for 12 weeks. Control group = Usual activities of daily living	VO_2_ max Muscle strength	VO_2_ peak significantly increased in both training groups as compared to controls: WEX by 2.5 mL/kg/min (95 % CI 0.6 to 4.4) and GEX by 2.3 mL/kg/min (95 % CI 0.6 to 4.0). When compared to the control, both WEX and GEX increased hamstring strength: WEX by 6.3 kg (95 % CI 1.2 to 11.3) and GEX by 7.6 kg (95 % CI 2.9 to 12.2). GEX enhanced leg press strength by 15.5 kg (95% CI 5.7 to 25.3) in comparison to control. Latissimus dorsi pulldown strength was only increased with GEX.	WEX was well tolerated. It enhanced body composition, leg strength, and aerobic ability. Data suggest WEX as a useful exercise training substitute for GEX for individuals with coronary heart disease.
Reed, J.L. et al. [13] 2021	Effect of high intensity interval training, walking on cardiovascular system in patients with coronary artery disease.	High intensity interval training = Session of 45 min was given to the patients that consist of warm-up high intensity training and cool-down. Moderate to vigorous intensity continuous training = A session of 60 min was given that consist of 10–15 min of warm-up, 10–15 min aerobic exercises and 15 min cool-down. Nordic walking = Session of 60 min was given to the patients that included 15 min warm-up, 10–15 min walking and 15 min cool-down.	Functional capacity Beck Depression Inventory-II (BDI-II) Quality of life Heart QoL is a 14-item self-report questionnaire	There was a higher increase in 6 MWT distance (m) for NW (77.2–60.9) than HIIT (51.4–47.8) and MICT (48.3–47.3), according to a significant time–group interaction (*p* = 0.042). BDI-II considerably improved (HIIT: 1.4 3.7, NW: 1.6 4.0, MICT: 2.3 6.0 points, main effect of time *p* < 0.001). Values for the SF-36 and Heart QoL significantly improved (main effects of time: *p* < 0.05).	All exercise programs (HIIT, NW, and MICT) were well-attended, secure, and helpful for enhancing physical and emotional health in patients with CAD.
Marzolini, S. et al. [15] 2008	Determine the effect of aerobic and resistance training in patients with coronary artery disease.	Aerobic training group = 30 to 60 min of walking and jogging AT/RT1 and AT/RT3 = Within a 24-week period, attended 6 RT exercise training sessions during their regularly scheduled weekly classes, including follow-up sessions at weeks 12, 16, and 22	Heart rate Muscle strength VO_2_ max	The average increase in VO_2_ peak from baseline was 11% for AT (*p* < 0.05), 14% for AT/RT1, and 18% for AT/RT3, although there was no statistically significant difference between the groups. VAT dramatically increased in the AT/RT3 group (*p* < 0.05). Only the AT + RT groups showed a decrease in body fat (*p* < 0.05). In comparison to AT alone, endurance improved greater in the AT + RT groups (*p* < 0.05). Compliance with the number of sets completed was less in than AT/RT1 is AT/RT3 (*p* < 0.02).	Despite of 28% reduction in the actual AT training stimulus, combination of RT and AT result in larger improvements in cardiovascular endpoints of exercise performance, skeletal muscle function, and body composition than AT alone. Findings strongly suggest use of multiple-set RT for patients requiring a higher RT stimulus and a combined training intervention in CAD patients.
Santa-Clara, H. et al. [16] 2002	The effect of a combined effect of aerobic and weight training program of 1 year in patients with coronary artery disease.	Aerobic training group = Session of aerobic exercises for 50 min along with warm-up and cool-down phase. Combined exercise group = Aerobic activities along with weight training program. Control group = No intervention	Peak oxygen consumption Heart rate Muscle strength VO_2_ max	One-repetition maximum approach was used for each of the eight weight exercises to measure muscle strength exclusively in the combined exercise group. Strength in the arms and legs rose from pre- to post-tests by 21.9 and 27.8 percent, respectively (*p* < 0.0001).	VT was raised more by weight training in addition to aerobic exercise than by aerobic exercise alone. When compared to the no-exercise group, aerobic exercise, whether it included weight training or not, significantly increased VO_2_, peak, functional capacity, and VT.
Kambic, T. et al. [17] 2021	The efficacy of high and low load resistance exercises on hemodynamic in patient with coronary artery disease.	Group 1 = Low load resistance exercises after 48–72 h rest then given high load resistance exercises. Group2 = High load resistance exercises after 48–72 h rest then given low load resistance exercises.	Heart rate Blood pressure Blood oxygen saturation Muscle strength Aerobic capacity	During HL-RE or LL-RE, no clinically significant changes in HR, BP, or patient-reported symptoms were noted. When compared to baseline, HR and SBP increased during LL-RE (from 66 to 86 bpm; from 129 to 146 mmHg; time effect: *p <* 0.001; and HL-RE (from 68 to 86 bpm; from 130 to 146 mmHg; time effect: *p* < 0.001). The increase in HR was larger after the final set of LL-RE (32 percent vs. 28 percent, *p* = 0.015) than it was after HL-RE.	Both HL-RE and LL-RE were effective and well-tolerated in patients with CAD.
Caruso, F.R. et al. [18] 2017	Evaluate the after effect of resistance exercises in patients with coronary artery disease on hemodynamic, autonomic, ventilatory, and metabolic alterations.	Aerobic group = Cardiovascular rehabilitation program that consist of aerobic exercises, stretching, treadmill training for 50 min 2 times a week for 8 weeks. Combined group = Cardiovascular rehabilitation program along with resistance training program. Each session was of 40 min 2 times a week for 8 weeks.	Heart rate Cardiac output Modified Baecke Questionnaire	RT increased maximal and submaximal load tolerance (*p* < 0.01), reduced hemodynamic response (*p* < 0.01), and decreased blood lactate levels during the 45° leg press. During exercise on a cycle ergometer and a 45° leg press, during the 8-week RT program increased parasympathetic tone and increased the SDNN index (*p* < 0.05).	An 8-week program of resistance exercise combined with aerobic exercise may reduce hemodynamic stress and alters metabolic and autonomic responses and reflect significant benefits on heart and autonomic nervous system.
Ximenes, N.N. et al. [19] 2015	Effect of early resistance exercises in patients with coronary artery disease.	Intervention group = Patients were provided with diaphragmatic breathing, resistance exercises and ambulation exercises 30 min twice a day. Control group = Patients were given diaphragmatic breathing exercises for 30 min twice a week.	Pulmonary function test Six-minute walk test	When compared to the control group, resistance exercise had no impact on the pulmonary function of the intervention group.	Early implementation of RT in CAD disease patient’s artery surgery is more beneficial and does not alter pulmonary function.
Deka, P. et al. [20] 2022	The effect of high intensity interval and resistance exercise training in patients with coronary artery disease.	Intervention group = In this group high intensity interval training along with resistance training of 50–60 min, 1 session per week for 8 weeks were given. Usual care group = Conventional medical treatment was given in this group to the patients.	International Physical Activity Questionnaire (IPAQ) 36-Item Short Form Health Survey (SF-36) questionnaire Functional capacity Systolic blood pressure Diastolic blood pressure Body composition	In comparison to the usual care group, there was a significant group and time interaction for the participants in the HIIT + R Group for BMI (*p* < 0.001), body fat percentage (*p* < 0.001), waist circumference (*p* = 0.001), physical activity (*p* < 0.001), functional capacity (*p<* 0.001), and QoL (*p* < 0.001). The HIIT + R group experienced a significant decrease in systolic blood pressure (*p* < 0.001).	To achieve desired health goals, elderly with CAD may benefit from a combined HIIT + R exercise strategy.
Arthur, H.M. [21] 2007	Effect of aerobic and combined aerobic strength training in women after an event of coronary artery by-pass grafting as post-surgery cardiac rehabilitation.	Intervention group = Aerobic exercises and aerobic plus strength training for a period of 6 months, 2 times in a week. Usual care group = Aerobic exercises for a duration of 6 months for 2 times in a week.	Short Form Health Survey (SF-36) Physical Component Summary Score (PCS) Exercise capacity Strength Self-efficacy	Both groups demonstrated statistically significant improvements in physical quality of life (*p* = 0.0002), peak VO2 (19% in aerobic/strength training vs. 22% in aerobic training alone), strength (*p =* 0.0001), and self-efficacy for stair climbing (*p* = 0.0024), lifting (*p* = 0.0001), and walking (*p* = 0.0012) after 6 months of supervised exercise training. Aerobic/strength training group had a statistically significant change in physical quality of life at the 1-year follow-up (*p* = 0.05).	Aerobic training alone and both aerobic/strength training are both beneficial in women with coronary artery disease. Combining strength and aerobic exercise maintains physical quality of life.
Schmid, J.P. et al. [22] 2007	The effect of endurance and resistance exercises after myocardial infarction in patients with coronary artery disease.	Combined group = endurance training and resistance training was given to patients for 4 times and 2 times respectively in a week. Endurance training group = In these group 6 sessions per week of endurance training of lower limb and abdominal muscles were given to the patients.	Muscle strength VO_2_ max	Over the course of a year, the end-diastolic volume increased from 206 ± 41 to 210 ± 48 mL (*p* = 0.379) compared to 183 ± 44 to 186 ± 52 mL (*p* = 0.586), the Left Ventricular mass increased from 149 ± 28 to 155 ± 31 g (*p* = 0.408) compared to 144 ± 36 to 149 ± 42 g (*p* = 0.227), and peak oxygen consumption and muscle strength both increased dramatically in both groups.	For three months, either an ET/RT combination or an ET alone can dramatically raise peak VO_2_ and muscle strength.
Pimenta, N.M. et al. [23] 2013	Effect of combined exercise training on body fat response in male coronary artery disease patients.	Combined exercise training group = Aerobic training and resistance training on 3 non-consecutive days in a week for 12 months. Control group = Aerobic training was given to the patients.	Trunk extremity fat ratio Aerobic capacity Muscle strength VO_2_ max Body composition	Both groups’ body mass and BMI did not differ between the start of the study and the end of the follow-up, but in the CET group, a significant decrease in all the outcome measures were observed.	Despite no changes in body mass or BMI, CET has positive impact on the body composition in CAD patients. No changes in BF distribution, which indicates that the rate of fat loss was identical across all BF depots that were evaluated.

Abbreviations: VO_2_ = Maximum Oxygen Consumption, RMSSD = Root Mean Square of Successive Difference, SD = Standard Deviation, SDNN = Standard Deviation of Normal Intervals UC = Usual Care, MWT = Minute Walk Test, AT—Aerobic Training, RT = Resistance Training, BMI = Body Mass Index, VT = Ventilatory Threshold, LV = Left Ventricle, ET = Endurance Training, HL-RE = High Load Resistance Exercise, LL-RE = Low Load Resistance Exercise, HR = Heart Rate, SB P= Systolic Blood Pressure, WEX = Water based Exercise, GEX = Gym based Exercise, CET = Combined aerobic and resistance Training, HIIT + R = High Intensity Interval Training+ Resistance, MICT = Moderate Intensity Continuous Training, NW = Nordic Walking BF = Body Fat, BP = Blood Pressure, HIIT = High Intensity Interval Training.

**Table 3:** Methodological quality assessment via PEDro scale.

Criteria *												
Study Authors/Year	1	2	3	4	5	6	7	8	9	10	11	Score
Hansen, D. et al. [10] 2011	1	1	1	1	-	-	-	1	-	1	1	6/10
Caruso, F.R. et al. [11] 2015	1	1	1	1	-	-	-	1	1	1	1	7/10
Scheer, A. et al. [12] 2021	1	1	1	1	-	-	-	1	1	1	1	8/10
Reed, J.L. et al. [13] 2021	1	1	1	1	-	-	-	1	1	1	1	8/10
Marzolini, S. et al. [15] 2008	1	1	-	1	-	-	-	1	-	1	1	5/10
Santa-Clara H. et al. [16] 2002	1	-	-	1	-	-	-	1	-	1	1	5/10
Kambic, T. et al. [17] 2021	1	1	1	1	-	-	-	1	-	1	1	7/10
Caruso, F.R. et al. [18] 2017	1	1	1	1	-	-	-	1	-	1	1	7/10
Vysoký, R. et al. [24] 2015	1	1	1	1	-	1	-	1	-	1	1	7/10
Ximenes, N.N. et al. [19] 2015	1	1	-	1	-	-	-	1	-	1	1	6/10
Deka, P. et al. [20] 2021	1	1	1	1	-	-	1	1	-	1	1	8/10
Arthur, H.M. et al. [21] 2007	1	1	1	1	-	1	-	1	1	1	1	9/10
Schmid, J.P. et al. [22] 2008	1	1	-	1	-	-	-	1	-	1	1	6/10
Pimenta, N.M. et al. [23] 2013	1	1	-	1	-	-	-	1	-	1	1	6/10

Criteria *—1. Specific eligibility requirements were followed 2. Subjects were randomly divided into groups. 3. The allocation was concealed. 4. The most crucial prognostic factors were identical across the groups at inception 5. All participants were blinded 6. All therapists who delivered the therapy were blinded 7. All assessors who measured at least one important outcome were blinded 8. More than 85% of the subjects who were initially divided into groups provided measurements of at least one major outcome 9. Analysis with the intention to treat 10. Comparison between groups 11. Study provides measures of variability. Each favorable outcome in a study receives a score of 1 on a scale from 0 to 10.

## Data Availability

All relevant data supporting this study’s finding are within the manuscript.
